# Primary Pulmonary Synovial Sarcoma in Pregnancy

**DOI:** 10.1155/2012/326031

**Published:** 2012-11-07

**Authors:** K. Bunch, S. H. Deering

**Affiliations:** Department of Obstetrics and Gynecology, Madigan Army Medical Center, Tacoma, WA 98431, USA

## Abstract

*Background*. Primary pulmonary synovial sarcoma is a rare malignancy with a poor prognosis. Surgical resection and postoperative management of these tumors has not been previously described in pregnancy. *Case*. A 38-year-old pregnant woman was admitted for evaluation of a right thoracic mass found on chest radiography at 26 weeks of gestation. A computed tomography-guided biopsy was subsequently completed and demonstrated a high-grade neoplasm. A right pneumonectomy was performed at 28 weeks of gestation due to pulmonary decompensation, and pathological examination revealed a pulmonary synovial sarcoma. The patient developed a postpartum pulmonary embolism and expired 6 weeks after delivery. *Conclusion*. Aggressive intervention for pulmonary malignancies during pregnancy may be necessary. Complete tumor resection is the most important prognostic factor in primary pulmonary synovial sarcoma.

## 1. Introduction

Soft-tissue synovial sarcomas commonly arise in paraarticular locations of the extremities but are rarely found in the lungs. Primary pulmonary synovial sarcoma is an aggressive tumor accounting for approximately 0.05% of all primary lung malignancies [1, 2]. The most common presenting symptoms include chest pain, cough, dyspnea, and hemoptysis. Diagnosis is made by a combination of radiologic imaging, histologic analysis, and cytogenetic testing. The recent identification of a chromosomal translocation specific to pleuropulmonary synovial sarcoma has increased the recognition of this particular sarcoma subtype. The chromosomal translocation t(x;18) is present in more than 80% of cases of primary pulmonary synovial sarcoma [3]. The mainstays of treatment are surgical resection followed by radiation or chemotherapy.

## 2. Case Presentation

A 38-year-old African American woman at 26 weeks' gestation presented with a 3-week history of dyspnea, upper right back pain, and orthopnea. Her pregnancy was complicated by the finding of a small fetal ventricular septal defect on ultrasound. The remainder of her medical history was noncontributory. Physical examination revealed a patient in moderate distress with a heart rate of 126, blood pressure 140/68, and respiratory rate of 25. Auscultation was notable for crackles in the base of the right lung. Chest roentgenography demonstrated complete opacification of the right lower lobe with an adjacent effusion. 

**Figure 1:**
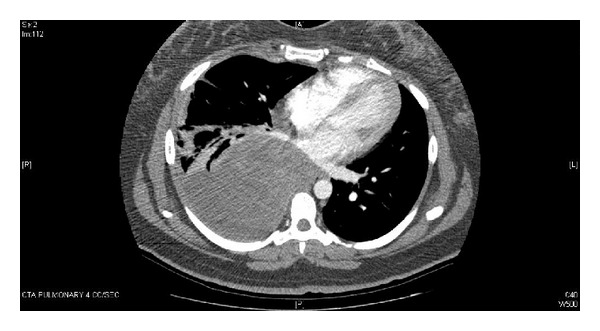
Helical CT with contrast demonstrating a large partially calcified mass within right hemithorax with broad-base apposition to mediastinum (24DEC2009).

A computed tomography (CT) angiogram was performed revealing a large, 12 cm partially calcified mass in the right chest with broad-based apposition to the mediastinum. This was followed by a CT-guided needle biopsy demonstrating a high-grade neoplasm (Figure 1). The patient began to experience acutely worsening dyspnea and chest pressure at 28 weeks' gestation and surgical management was pursued to optimize her pulmonary status. After antenatal corticosteroid administration, a right pneumonectomy and lymph node dissection were performed; however, negative margins were not obtained due to the extent of tumor burden. The final pathology confirmed the diagnosis of a high-grade, poorly differentiated pulmonary synovial sarcoma. A multidisciplinary team was assembled to include pulmonology, medical and radiation oncology, cardiothoracic surgery, and high-risk obstetrics. Given the patient's stable clinical status and the extremely preterm gestational age, the decision was made to proceed with administration of corticosteroids for fetal lung maturity in preparation for possible preterm delivery for worsening maternal status.

The patient was followed closely with antepartum fetal testing and serial growth sonography. Intrauterine growth restriction was diagnosed at 30 weeks' gestation with an estimated fetal weight at the 6-percentile. Due to subsequent poor interval growth, a repeat cesarean section was performed at 32 weeks' gestation. The patient had an uncomplicated postoperative course and was discharged home on hospital day 3. Following delivery the newborn developed cardiac decompensation and was transferred for a pulmonary artery banding procedure. The infant was discharged to home on day of life 50. 

**Figure 2:**
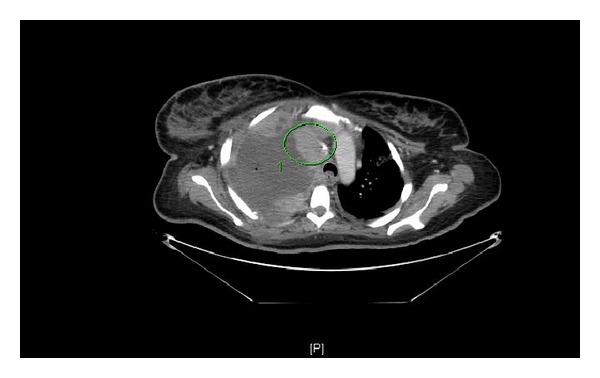
CT with contrast demonstrating near-complete superior vena cava occlusion by tumor invasion/extrinsic mass effect (21MAR2010).

Four weeks after delivery, the patient re-presented with acute shortness of breath and was diagnosed with a pulmonary embolus. A repeat CT scan revealed continued tumor growth and near complete occlusion of the superior vena cava by extrinsic mass effect and tumor invasion (Figure 2). The patient received radiation therapy upon readmission, however, due to worsening hepatic function, the patient was unable to start chemotherapy. The patient required continued care in the intensive care unit, prolonged ventilation, and tracheostomy placement. She continued to deteriorate and expired six weeks after delivery due to cardiopulmonary arrest and hepatic failure. 

## 3. Discussion

### 3.1. Clinical Discussion

Primary pulmonary synovial sarcoma (PPSS) is a rare malignancy with few reported cases and only one other case in pregnancy [4]. Primary pulmonary sarcomas are exceedingly rare, accounting for approximately 0.05% of all primary lung malignancies, and less than 10% of all soft tissue sarcomas. Pulmonary synovial sarcomas arise in lung, pleura, chest wall, and mediastinum [2, 3]. The most frequently reported pulmonary sarcomas include fibrosarcoma, leiomyosarcoma, and malignant fibrous histiocytoma. Pulmonary synovial sarcoma has become an increasingly recognized sarcoma subtype due to recent identification of a distinct chromosomal translocation specific to synovial sarcoma. Typical presenting symptoms include dyspnea (8–36%), chest pain (24–80%), cough (10–33%), and hemoptysis (20–25%). Less commonly reported symptoms include shoulder or back pain, fever, and extremity swelling [4, 5]. Differential diagnoses include nonmalignancies such as granulomatous disease, rheumatoid nodules, and pyogenic abscess, to malignancies such as metastatic lesions, bronchogenic carcinoma, adenocarcinomas, lymphoma, carcinosarcomas, among others [5, 6]. 

### 3.2. Pathologic and Radiographic Discussion

Diagnosis is made by histologic appearance, immunohistochemistry, and more recently cytogenetic analysis. Cytogenetic analysis has become an increasingly important tool in diagnosing pulmonary synovial sarcoma. Primary pulmonary synovial sarcoma is characterized by a reciprocal chromosomal translocation t(x;18)(p11;q11) which fuses the *SYT *gene on chromosome 18 to one of two homologous genes on chromosome x, *SSX1* or *SSX2. *The fusion transcripts are detected by reverse transcriptase-polymerase chain reaction (RT-PCR) or fluorescence in situ hybridization (FISH) and are thought to function as aberrant transcription regulators [2, 6]. These distinct chromosomal translocations appear to be specific and are present in 80–90% of synovial sarcomas [4, 7]. 

The radiologic features of PPSS vary and overlap with many other pulmonary and pleural lesions. On chest radiograph various descriptions have been reported to include focal pleural thickening, opacification of a hemithorax, consolidation, and uniform or homogeneous masses with well-circumscribed borders. An ipsilateral pleural effusion may be present; however, pneumothorax, cavitation, and calcification are not typically described. In our patient, the initial appearance was that of a pleural effusion.

On CT, PPSS is often well-defined heterogeneous masses containing areas of fluid attenuation compatible with hemorrhage or necrosis. Tumor calcification, infiltration of the chest wall, and cortical bone destruction may be demonstrated with synovial sarcomas that arise in the chest wall [5, 8]. Magnetic resonance imaging (MRI) has been described in the literature primarily in cases of chest wall masses. MR imaging often demonstrates dramatic heterogeneity including internal fluid-fluid levels corresponding with hemorrhage, multilocular components, and further enhancement with gadolinium contrast, not generally seen on CT. The “triple sign” (bright, dark, and gray) corresponding with tumor, hemorrhage, and necrosis on MRI is often described in regards to PPSS [5]. With PPSS the lack of prominent vascular structures on MR imaging, tumor calcifications and bone involvement noted on CT are some distinct differences compared to soft-tissue synovial sarcomas. In addition, adenopathy is not a feature of PPSS and the presence of adenopathy on imaging favors lung cancer [4, 9]. 

### 3.3. Pregnancy-Related Concerns

Given the rarity of this malignancy and the dearth of data regarding the natural history of the disease, there are no guidelines directing optimal treatment, particularly in pregnancy. Furthermore, no controlled studies investigating adjuvant chemotherapy have been undertaken for such reasons. There is no standardized therapy; however, current standards of treatment involve multimodal therapy including surgical resection followed by adjuvant chemotherapy, radiotherapy, or both [4, 6]. In general, synovial sarcomas are chemosensitive to doxorubicin and ifosfamide with a reported response rate of 24–50% [2, 4]. The lack of standardized therapy and the concerns about adverse effects on the fetus with chemotherapy use make recommendations even more difficult during pregnancy. In consultation with the surgical oncologist and pulmonary critical care providers, we waited to perform surgery until the patient's worsening pulmonary symptoms declined to the point where resection was felt to be the best option and fetal outcome could be maximized if preterm delivery was necessary. The pregnancy was followed closely postoperatively with antepartum fetal testing and serial growth scans. In discussion with the family, it was important to balance treatment of the patient's cancer with the gestational age and fetal condition.

The overall reported 5-year survival rate for this type of malignancy is approximately 50%. Predictors of poor prognosis include male gender, age over 20 years, tumor size >5 cm, large number of mitotic figures (>10 per high-powered field), poorly differentiated tumor cells, high grade, and neurovascular invasion. The most important prognostic factor is complete tumor resection. Metastases are common with tumors greater than 10 cm in diameter [2, 3]. In our patient, she had several predictors of poor prognosis to include age, tumor size, high mitotic rate, and incomplete tumor resection.

## 4. Conclusion

This case highlights not only the diagnostic and treatment dilemma for PPSS in pregnancy, but also the poor prognosis associated with PPSS. Malignancies, even those as rare as PPSS, must be considered in the differential diagnosis of a pulmonary mass in pregnancy. Characteristic findings on computed tomography or magnetic radiography may help further to clarify the diagnosis. Given the aggressive nature of this tumor, early intervention during pregnancy with tumor resection and addition of chemotherapy treatment should be considered to optimize maternal outcomes. However, the gestational age and fetal condition must also be evaluated when clarifying palliative options.
